# Comparative transcriptome analysis provides insights into the molecular mechanism underlying double fertilization between self-crossed *Solanum melongena* and that hybridized with *Solanum aethiopicum*

**DOI:** 10.1371/journal.pone.0235962

**Published:** 2020-08-06

**Authors:** Dandan Li, Siqi Li, Wenjia Li, Ake Liu, Yaqin Jiang, Guiyun Gan, Weiliu Li, Xuyu Liang, Ning Yu, Riyuan Chen, Yikui Wang

**Affiliations:** 1 College of Horticulture, South China Agricultural University, Guangzhou, China; 2 Institute of Vegetable Research, Guangxi Academy of Agricultural Sciences, Nanning, China; 3 Guangxi Key Laboratory of Vegetable Breeding and New Technology Development, Nanning, China; ICAR-Indian Institute of Agricultural Biotechnology, INDIA

## Abstract

Wild relatives represent a source of variation for many traits of interest for eggplant (*Solanum melongena*) breeding, as well as for broadening its genetic base. However, interspecific hybridization with wild relatives has been barely used in eggplant breeding programs, and reproductive barriers have resulted in reduced seed numbers in interspecific combinations. The mechanism underlying this phenomenon remains unclear. We hybridized females of cultivated eggplant 177 (*Solanum melongena*) with males of wild relatives 53 and Y11 (*Solanum aethiopicum*). Self-crossed 177 was the control. The seed number per control fruit was significantly higher than that of the hybrids. Paraffin sections showed no significant difference between control and 177×53 and 177×Y11. Double fertilization began 4 days post-pollination. Sperm cells were fused with egg cells 6 days post-pollination. To understand the differences in molecular mechanisms underlying this process, transcriptomes of ovaries at **0, 4, and 6 days** after self-crossing and hybridization were analyzed. We screened **22,311** differentially expressed genes (DEGs) between the control and hybrids 4 and 6 days post-pollination. A total of 497 DEGs were shared among all pollination combinations. These DEGs were enriched in plant hormone transduction, cell senescence, metabolism, and biosynthesis pathways. DEG clustering analysis indicated distinct expression patterns between the control and hybrids but not between the hybrids. The DEGs in hybrids involved secondary metabolic process, phenylpropanoid metabolic process, and carboxypeptidase activity, while those in the control involved xyloglucan metabolic process, auxin-activated signaling pathway, cell wall polysaccharide metabolic process, and xyloglucosyl transferase activity. Additionally, 1683 transcription factors, including members of the AP2-ERF, MYB, bHLH, and B3 families may play important roles in self-crossing and hybridization. Our results provide insights into the regulatory mechanisms underlying variations between ovaries of self-crossed and hybrid eggplants and a basis for future studies on crossbreeding *Solanum* and genetic mechanisms underlying double fertilization.

## Introduction

Eggplant belongs to the Solanaceae family and is an important vegetable worldwide especially in Africa and Southeast Asia [1]. It has great nutritional and medicinal value and is rich in proteins, minerals, and antioxidants. However, eggplant production is severely threatened by several soil-borne diseases and is prone to insect damage; these threats include fusarium wilt, verticillium wilt, and root-knot nematode [2, 3]. Some *Solanum* species that are related to eggplant, such as *Solanum aethiopicum*, *Solanum linnaeanum*, *Solanum sisymbriifolium*, *Solanum aculeatissimum*, and *Solanum torvum*, have disease resistance that makes them useful for eggplant breeding [4, 5]. Therefore, the transfer and introgression of useful resistance genes into eggplant from diverse sources is important because of the lack of resistance in the common eggplant gene pool, which is a limitation to major breeding advances [6]. Thus, crossbreeding eggplant with its wild relatives is a very effective way to broaden its genetic background and improve cultivated eggplant resistance [7]. However, owing to different genetic backgrounds, the effective utilization of wild relatives is seriously hindered by reproductive obstacles in the process of distant hybridization [8]. Previous studies have reported that, for the interspecific hybridization of eggplant, F1 generations were either not available or only a small number were available [5, 9–11] and some of these were sterile [12, 13]. The number of F1 generations obtained is related to the efficiency of double fertilization and mutual recognition of male and female gametes to form zygotes, which was reported as the key to F1 production [14].

There are many studies on the mechanism of double fertilization, which showed that double fertilization is a very complex physiological activity involving different molecular functions and metabolic pathways. Multiple plant hormones [15–17], transcription factors [18], and family genes [19] regulate double fertilization and seed development. For example, overexpression of the gibberellin oxidase genes *PsGA2ox2* (gibberellin inactivation gene) and *SlGA2ox1* in *Arabidopsis thaliana* and *Solanum lycopersicum* resulted in decreased ovules and aborted seeds [20, 21]. Although many key genes and functions in double fertilization have been reported [22, 23] on model plants and crops such as *Arabidopsis* and rice [24, 25], there have been no reports on eggplant to our knowledge.

In preliminary research, we found that interspecific hybridization produced relatively few seeds and there was a significant difference in the seed number per fruit in different interspecific combinations (unpublished). We wished to understand better the difference in seed number and study the molecular mechanism of interspecific hybridization, which will help to improve eggplant breeding. In the present study, we compared seed numbers between self-crossed and hybridized eggplants, paraffin sections were examined to determine the differences in double fertilization over time, and RNA-Seq was finally used to compare the expression profiles of self-crossed and hybridized offspring during double fertilization. Our study aimed to reveal the underlying mechanism causing the differences in fruit seed number, and our results may provide important information for *Solanum* crossbreeding to develop further our understanding of the genetic mechanisms underlying the double fertilization process.

## Materials and methods

### Plant materials and pollination

*Solanum melongena* (177) and the wild-related species *Solanum aethiopicum* (53, Y11) were obtained from the Guangxi Academy of Agricultural Sciences. They were germinated on July 9, 2018; planted on September 6, 2018; and pollinated from October 11–31, 2018. Every plant breed had three plots with 100 plants per plot, which were arranged in random blocks. Field management was carried out in the usual way. The second to the eighth flowers were selected for pollination. The female parents were castrated before pollination. The test was carried out at the Lijian Research Base of Guangxi Academy of Agricultural Sciences.

### Seed numbers of single fruit

Pollen on the flowering day was selected for pollination and then labeled and bagged in a small paper package. Four days after pollination, the packages were removed and confirmed. The seed number and morphological characteristics were analyzed when the fruits were ripe.

### Observation of ovary paraffin sections

The ovary or fruit was sampled separately after 2, 4, 6, 8, and 10 days of artificial pollination and fixed with FAA (70% absolute ethanol: glacial acetic acid: formaldehyde = 90:5:5) and then stored in a refrigerator at 4 °C. The materials were dehydrated by ethanol gradient for 1.5 h per stage (70%, 85%, 95%, and 100% ethanol). After the material was thoroughly dehydrated, it was placed in a 1:1 mixture of xylene: absolute ethanol for 2 h and then pure xylene for 2 h (2 times). The transparent material was immersed in xylene:paraffin (1:1, 42 °C, 24 h) for embedding. The wax pieces of 10–12 μm thickness were sectioned and baked in a 45 °C oven for routine dewaxing (pure xylene and 1:1 xylene:paraffin). The sections were rehydrated with different gradients of ethanol and dyed with 1% toluidine blue aqueous solution for 30–60 s. Then, the sections were dehydrated and mounted.

### RNA isolation and transcriptome sequencing

We collected the ovaries of unpollinated 177 and 177 × 177, 177 × 53, and 177 × Y11 4 and 6 days after pollination. They were frozen in liquid nitrogen and stored in a refrigerator at −80 °C until total RNA extraction. The RNA samples were initially assessed by 1% agarose gel electrophoresis for degradation and contamination. Qualified samples were quantified by a Qubit2.0 fluorometer and their integrity was assessed by using an Agilent 2100 Bioanalyzer. The ovaries of unpollinated 177, 177 × 177 4 days after pollination, 177 × 177 6 days after pollination, 177 × 53 4 days after pollination, 177 × 53 6 days after pollination, 177 × Y11 4 days after pollination, and 177 × Y11 6 days after pollination were used to construct the cDNA library, and were respectively named NP, AP1774D, AP1776D, AP534D, AP536D, APY114D, and APY116D. Technical services were provided by Major Biotechnology (China, Shanghai). Briefly, total RNA of the seven samples was extracted (Invitrogen, Carlsbad, CA, USA) first and eukaryotic mRNA was enriched with oligo(dT) magnetic beads. Then, fragmentation buffer was used to break the mRNA into short fragments. Using the fragmented mRNA as a template, the first cDNA strand was synthesized with random hexamers. Then, the buffer, dNTPs, RNase H, and DNA polymerase I were added to synthesize the second cDNA strand. After purification using a QiaQuick PCR kit and elution with the supplied EB buffer, terminal repair was carried out, poly(A) was added with the sequencing adapter, and the paired-end RNA-Seq sequencing library was sequenced with an Illumina Novaseq 6000 System (2×150 bp read length).

### Assembly and annotation of sequencing data

The raw paired-end reads were trimmed and subjected to quality control using SeqPrep (https://github.com/jstjohn/SeqPrep) and Sickle (https://github.com/najoshi/sickle) with default parameters. Then, the clean reads were separately aligned to the reference genome (PRJNA612792) with orientation mode using TopHat (http://tophat.cbcb.umd.edu/, version 2.1.1) software. To identify differentially expression genes (DEGs) between two different samples, the expression level of each transcript was calculated according to the fragments per kilobase of exon per million mapped reads (FPKM) method. RSEM (http://deweylab.biostat.wisc.edu/rsem/) [26] was used to quantify gene abundance. R statistical package software EdgeR (Empirical analysis of Digital Gene Expression in R, http://www.bioconductor.org/packages/2.12/bioc/html/edgeR.html) [27] was utilized for differential expression analysis. In addition, functional-enrichment analysis including Gene Ontology (GO) and Kyoto Encyclopedia of Genes and Genomes (KEGG) analyses were performed to identify which DEGs were significantly enriched in GO terms and metabolic pathways at a Bonferroni-corrected P-value of ≤0.05 compared with the whole-transcriptome background. GO functional enrichment and KEGG pathway analysis were performed using Goatools (https://github.com/tanghaibao/Goatools) and KOBAS 2.1.1 (http://kobas.cbi.pku.edu.cn/download.php) [28]. The homologous transcription factor information was obtained through HMMER software analysis or compared with PlantTFDB (http://planttfdb.cbi.pku.edu.cn/).

### Differentially expressed unigene analysis and functional enrichment analysis

The seven samples (AP1774D, AP1776D, AP534D, AP536D, APY114D, APY116D, and NP) were used with NP as a blank control. AP1774D and APY116D were used as test data of 4 days and 6 days post-pollination, respectively. P-adjust < 0.05 & |log2FC| ≥ 1 were used as parameters and the DEGs were statistically analyzed by DESeq2 software; the method of multiple test correction was BH (false discovery rate (FDR) correction with Benjamini/Hochberg). GO enrichment analysis was performed on DEGs using Goatools software; Fisher’s exact test was used for comparisons and a corrected P-value (P-adjust) < 0.05 was considered significant. KEGG pathway enrichment analysis was performed on the genes/transcripts by using the independent research and development process of Majorbio; Fisher’s exact test was used for comparisons and an uncorrected P-value < 0.05 was considered significant.

### Quantitative reverse transcription PCR analysis

Total RNA was extracted using a total RNA extraction kit (DP419, Tiangen Biochemical Technology). Reverse transcription was performed using the TransScript^®^ One-Step gDNA Removal and cDNA Synthesis SuperMix Kit purchased from TransGene Biotech. Amplifications and fluorescence detection were performed on the LightCycler 480 System (Roche, Basel, Switzerland) using the TransStart^®^ Tip Green qPCR SuperMix. Three biological replicates and three technical replicates were used per reaction. qRT-PCR was performed under the following conditions: 95 °C for 3 min; 40 cycles at 95 °C for 15 s per cycle, and 58 °C for 30 s. The DNA primers are listed in S1 Table. The relative expression of each gene was calculated using the 2^−ΔΔCt^ method, and actin was employed as a reference.

## Results

### Comparing seed number and morphology among different pollination combinations

To investigate the reproductive incompatibility between *S*. *melongena* and *S*. *aethiopicum*, we statistically analyzed the differences among the F1 generations of different hybridization combinations (Table 1). The seed number per fruit in the self-crossed plants was significantly higher than in the hybrids. The average length, average length/width, and thousand-grain weight of the hybrid F1 seeds were significantly greater than those of the self-crossed seeds. The longest average length was observed in the 177 × 53 seeds (2.73 mm), which was significantly longer than that of the 177 × Y11 (2.65 mm) and self-crossed 177 (2.50 mm) seeds. The average width of the 177 × 53 seeds (2.15 mm) was significantly higher than that of the 177 × Y11 seeds (2.08 mm) but not significantly different from that of the 177 × 53 seeds (2.19 mm). These results indicate that different hybridization combinations were highly compatible. However, differences remained between the self-crossed and hybridized seeds.

**Table 1:** Seed number and morphological characteristics.

Pollination combination	Number of seeds per fruit	Thousand seed weight (g)	Average Length/width	Average Length (mm)	Average Length (mm)
**177**	784 ± 8^aA^	5.60 ± 0.12^bA^	1.21 ± 0.01^bA^	2.50 ± 0.02^cB^	2.08 ± 0.04^bA^
**177 × 53**	170 ± 12^cB^	6.09 ± 0.06^aA^	1.25 ± 0.01^aA^	2.73 ± 0.02^aA^	2.19 ± 0.02^aA^
**177 × Y11**	215 ± 13^bB^	6.02 ± 0.06^aA^	1.24 ± 0.01^aA^	2.65 ± 0.02^bA^	2.15 ± 0.01^abA^

Different lowercase letters in the same column mean significant difference at P < 0.05, while different uppercase letters in the same column mean significant difference at P < 0.01.

**Fig 1:**
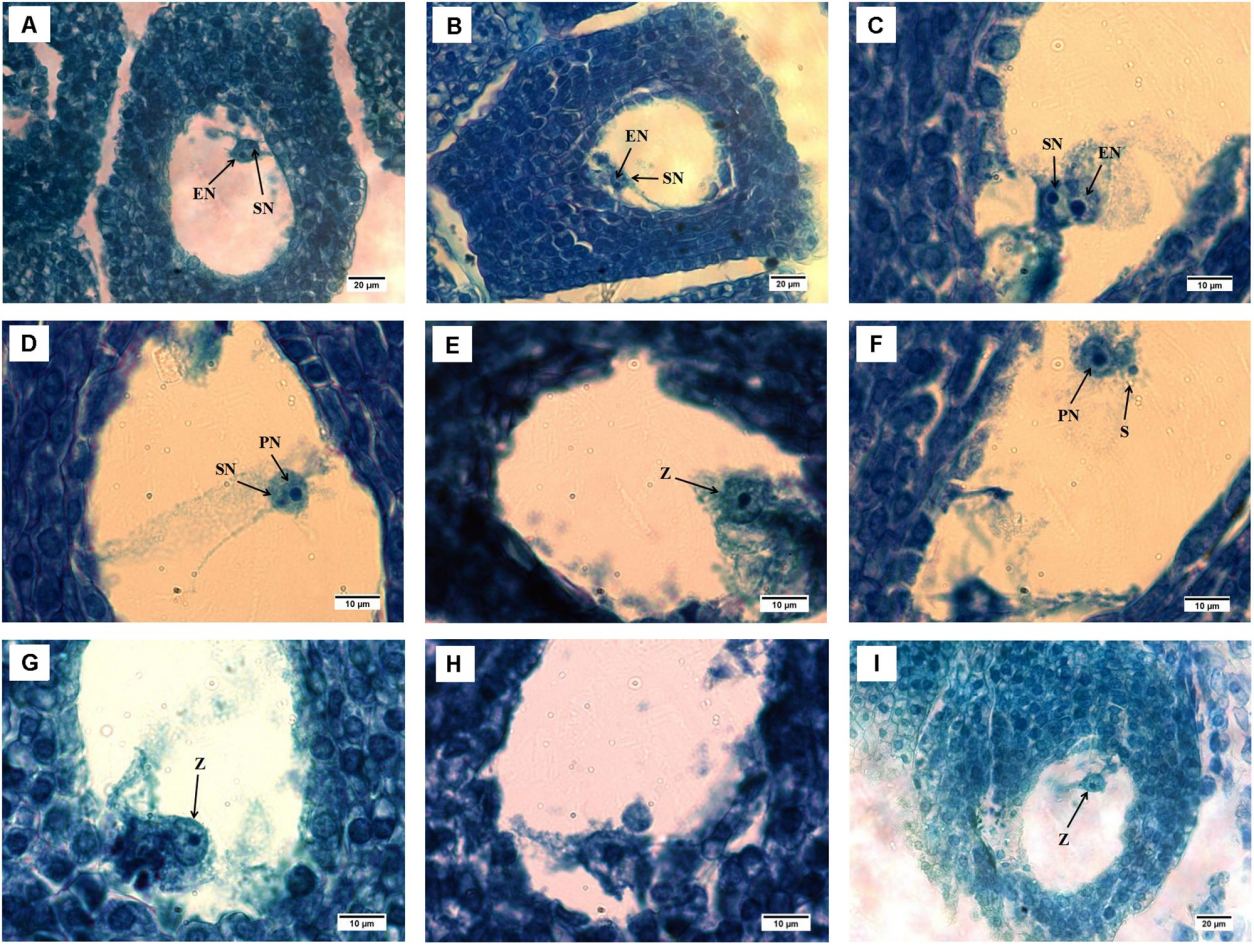
Stained paraffin sections of the double fertilization process. A. 177 sperm nucleus is close to the egg nucleus 4 days after pollination; B. 177 × 53 sperm nucleus is close to the egg nucleus 4 days after pollination; C. 177 × Y11 sperm nucleus is close to the egg nucleus 4 days after pollination; D. 177 spermatic nucleus is close to the polar nucleus 6 days after pollination; E. 177 × 53 sperm cells are close to the polar nucleus 6 days after pollination; F. 177 × Y11 sperm cells are close to the polar nucleus 6 days after pollination; G. 177 sperm and egg nuclei are fused 6 days after pollination; H. 177 × 53 sperm and egg nuclei are fused 6 days after pollination; I. 177 × Y11 sperm and egg nuclei are fused. J. 177 primary endosperm nucleus enters a stage of division 10 days after pollination. E: Egg cell; Sy: Synergid cell; EN: Egg nucleus; SN: Sperm nucleus; PN: Polar nucleus; S: Sperm; Z: Zygote.

To study the reasons for these differences in seed number per fruit and morphology among different pollination combinations, paraffin sections were prepared to observe the double fertilization process. After 2 days of pollination, the pollen tube had not entered the embryonic sac, but subsidiary and egg cells could be seen. Four days after pollination, **177, 177 × 53, and 177 × Y11** had one spermatic nucleus entering the egg cell near the nuclear membrane and another spermatic nucleus entering the polar nucleus. Thus, double fertilization had begun (Fig 1). Six days after pollination, the spermatic nucleus had fused with the egg nucleus, and the nucleoli of the two polar nuclei had fused with the nucleoli of one spermatic nucleus. Thus, double fertilization was completed (Fig 1E–1H). The sperm and egg nuclei are fused 6 days after pollination in self-crossed 177 (Fig 1I). In 177 × 53 and 177 × Y11, double fertilization began 4 days after pollination and zygote formation occurred 6 days after pollination. The timing of double fertilization between different combinations was consistent.

### Comparative transcriptome analysis during double fertilization

**Fig 2:**
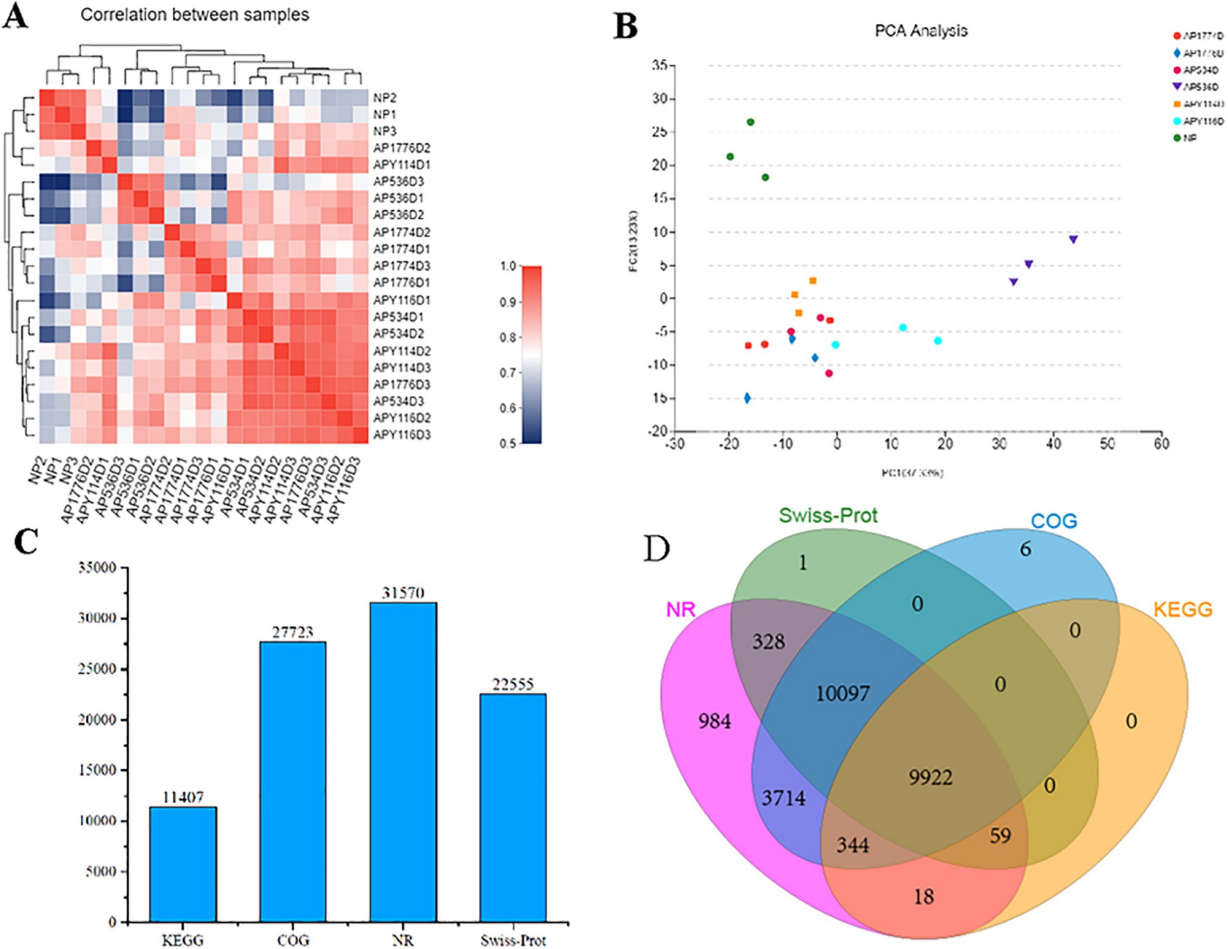
Basic information of sequencing data. A. pairwise Pearson’s correlation coefficients of sequencing data from seven samples with three replicates; B. principal component analysis (PCA) of transcriptomic variation. Colored dots represent different samples; C. annotation of gene numbers based on different databases; D. distribution of annotated genes on different databases.

To understand gene expression differences between self-crossed and hybrid seeds, ovaries were sampled at 0, 4, and 6 days after self-crossing and 4 and 6 days after hybridization of 177 × 53 and 177 × Y11 (respectively named NP, AP177-4D, AP177-6D, AP53-4D, AP53-6D, APY11-4D, and APY11-6D) for RNA-Seq analysis. The raw reads were qualified and adapters were removed, and 1,330,845,476 clean reads and 199,599,189,622 clean bases were obtained, with an average of 63,373,594 reads per sample. The Q30 was >94%, GC content was >43%, and error rate was 0.02% (S1 Table). RNA-Seq sequences were mapped on the eggplant assembly genome CNP0000734. Finally, 92.73%-97.7% of the reads were mapped to the genome regions (S2 Table). Pairwise Pearson’s correlation coefficients of the samples with their biological replicates indicated high repeatability of the sequencing data (Fig 2A). Except for AP53-6D, the expression profiles of the other samples were separated by self-crossing and hybridization. To obtain an overview of the transcriptomic variation, principal component analysis (PCA) was performed and the values of PC1 and PC2 were 20.6% and 9.1%, respectively (Fig 2B). The PCA clearly separated the three species into three clusters, suggesting the expression profiles of NP and AP53-6D were different from those of the other tissues. We annotated the DEGs based on four databases (Non-redundant Protein (NR), Swiss-Prot Protein Database (Swiss-Prot), Clusters of Orthologous Groups for Eukaryotic Complete Genomes (COG), and KEGG). As shown in Fig 2C and S3 Table, the NR database annotated the most genes at 31,570, and the KEGG, COG, and Swiss-Prot Pfam databases annotated 11,407; 27,723; and 22,555 genes, respectively.

**Fig 3:**
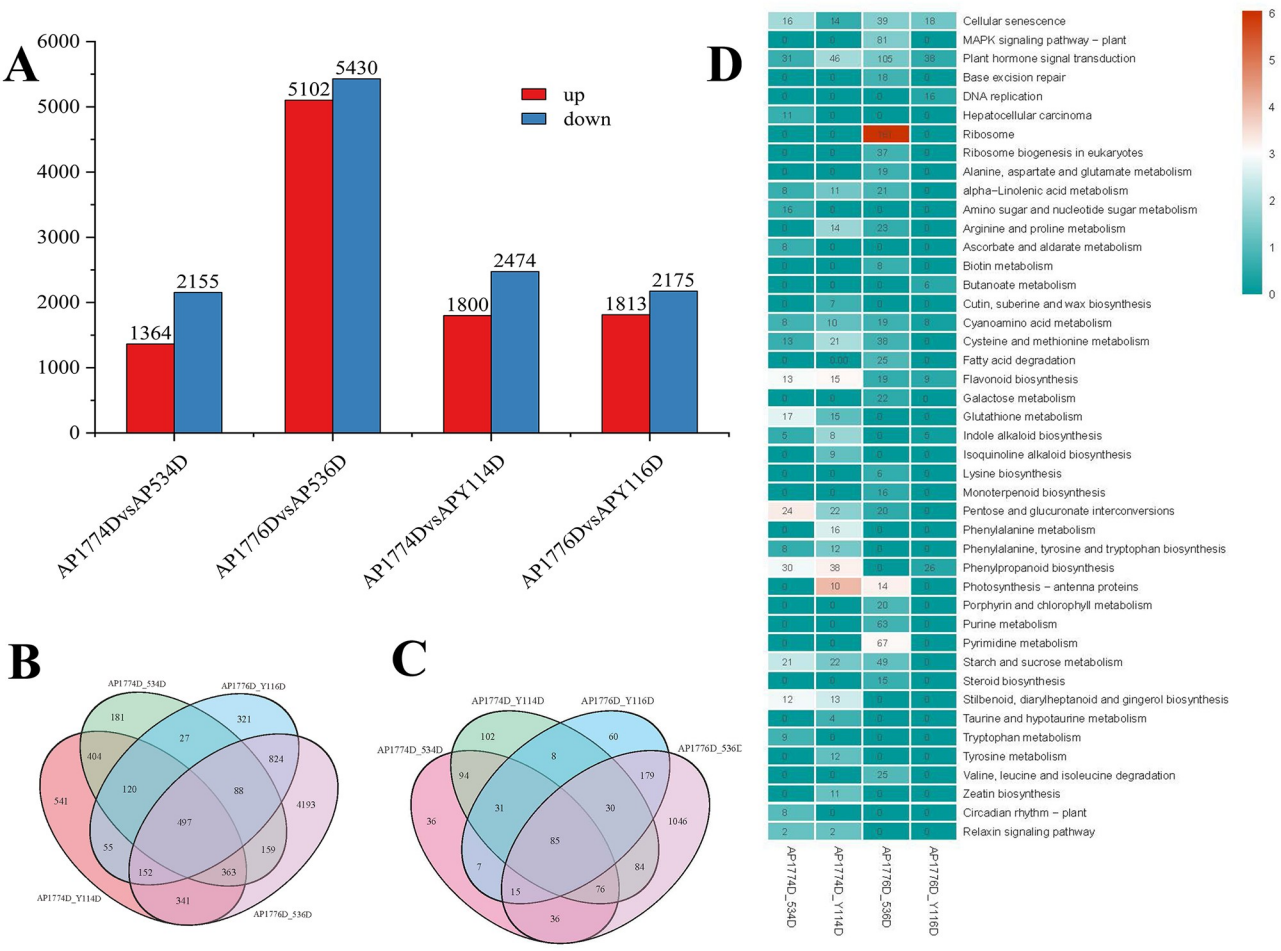
Comparative analysis of differentially expressed genes (DEGs). A. number of DEGs during self-fertilization and hybridization; B. comparison of DEGs during self-fertilization and hybridization; C. significantly enriched Kyoto Encyclopedia of Genes and Genomes (KEGG) pathway during self-fertilization and hybridization; D. heatmap of the number of DEGs in each KEGG pathway defined in the KEGG database.

To screen for DEGs between self-crossed and hybridized samples during double fertilization, NP was used as a blank control for the seven samples. We analyzed AP177-4D vs AP53-4D, AP177-4D vs APY11-4D, AP177-6D vs AP53-6D, and AP177-6D vs APY11-6D, with P <0.05 and |log2FC| ≥1 as the standard thresholds; 3519, 4272, 10532, and 3988 DEGs were obtained (Fig 3A), respectively. Among these DEGs, only 497 genes were differentially expressed in all four groups (Fig 3B). To study ovary gene expression patterns of self-crossed and hybridized samples over time, we selected DEGs 0, 4, and 6 days after pollination in self-crossed (AP177) and hybridized (APY11 and AP53) samples to analyze the expression modes at different times. Among them, there were 2983 DEGs in AP177, 7524 in AP53, and 4044 in APY11. We divided each group into 10 expression patterns. As shown in S1 Fig, most DEGs in AP177, AP53, and APY11 were in subcluster 5 (1255; 42.07%), subcluster 4 (3404; 45.24%), and subcluster 6 (2475; 61.2%), respectively. Furthermore, we conducted GO analysis on subcluster 5 (S2A Fig) of AP177, subcluster 4 (S2B Fig) of APY 53, and subcluster 6 (S2C Fig) of APY11. The results showed that the expression patterns of AP53 and APY11 were similar, while the expression patterns of AP53 and APY11 were different from that of AP177. The DEGs of AP53 and APY11 were mainly enriched in secondary metabolic process, phenylpropanoid metabolic process, and carboxypeptidase activity. The DEGs of AP177 were mainly enriched in xyloglucan metabolic process, auxin-activated signaling pathway, cell wall polysaccharide metabolic process, and xyloglucosyl transferase activity.

### Functional comparison of DEGs between self-crossed and hybrid ovaries

To clarify the functions of these genes (Fig 3A), we conducted GO and KEGG functional enrichment analysis (S3 and S4 Figs). Four days after pollination, the DEGs of AP177 vs AP53 and AP177 vs APY11 were significantly enriched in the regulation of cell cycle, cell wall, external encapsulating structure, pectin catabolic process, motor activity, polysaccharide catabolic process, microtubule-based process, microtubule binding, microtubule-based movement, and microtubule motor activity (FDR < 0.05). In addition, AP177 vs AP53 was significantly enriched in the DNA packaging complex, cellular polysaccharide metabolic process, cytoskeletal part, response to water, glucan catabolic process, carbon-oxygen lyase activity, pectate lyase activity, cellular glucan metabolic process, glucan metabolic process, and microtubule. AP177 vs APY11 was significantly enriched in photosynthesis, light harvesting in photosystem I, tubulin binding, movement of cell or subcellular component, photosynthesis, light harvesting, auxin-activated signaling pathway, drug catabolic process, carbohydrate catabolic process, cell wall organization, and extracellular region (S3A and S3B Fig). Six days after pollination, as shown in S3C and S3D Fig, the DEGs of AP177 vs AP53 and AP177 vs APY11 were significantly enriched only in supramolecular fiber, polymeric cytoskeletal fiber, supramolecular complex, and supramolecular polymer (FDR < 0.05). However, AP177 vs AP53 was also enriched in ribosome kinesin complex, cellular macromolecule biosynthetic process, non-membrane-bound organelle, macromolecule biosynthetic process, structural constituent of ribosome, organic substance biosynthetic process, cellular biosynthetic process, biosynthetic process, structural molecule activity, cellular nitrogen compound biosynthetic process, DNA-binding transcription factor activity, microtubule, tubulin binding, translation, and peptide biosynthetic process. AP177 vs APY11 was also significantly enriched in DNA replication initiation, origin recognition complex, regulation of catalytic activity, protein-DNA complex, regulation of protein kinase activity, regulation of protein phosphorylation, regulation of molecular function, microtubule-based movement, microtubule motor activity, regulation of phosphorylation, DNA replication, cell cycle process, nucleosome, movement of cell or subcellular component, and motor activity.

**Fig 4:**
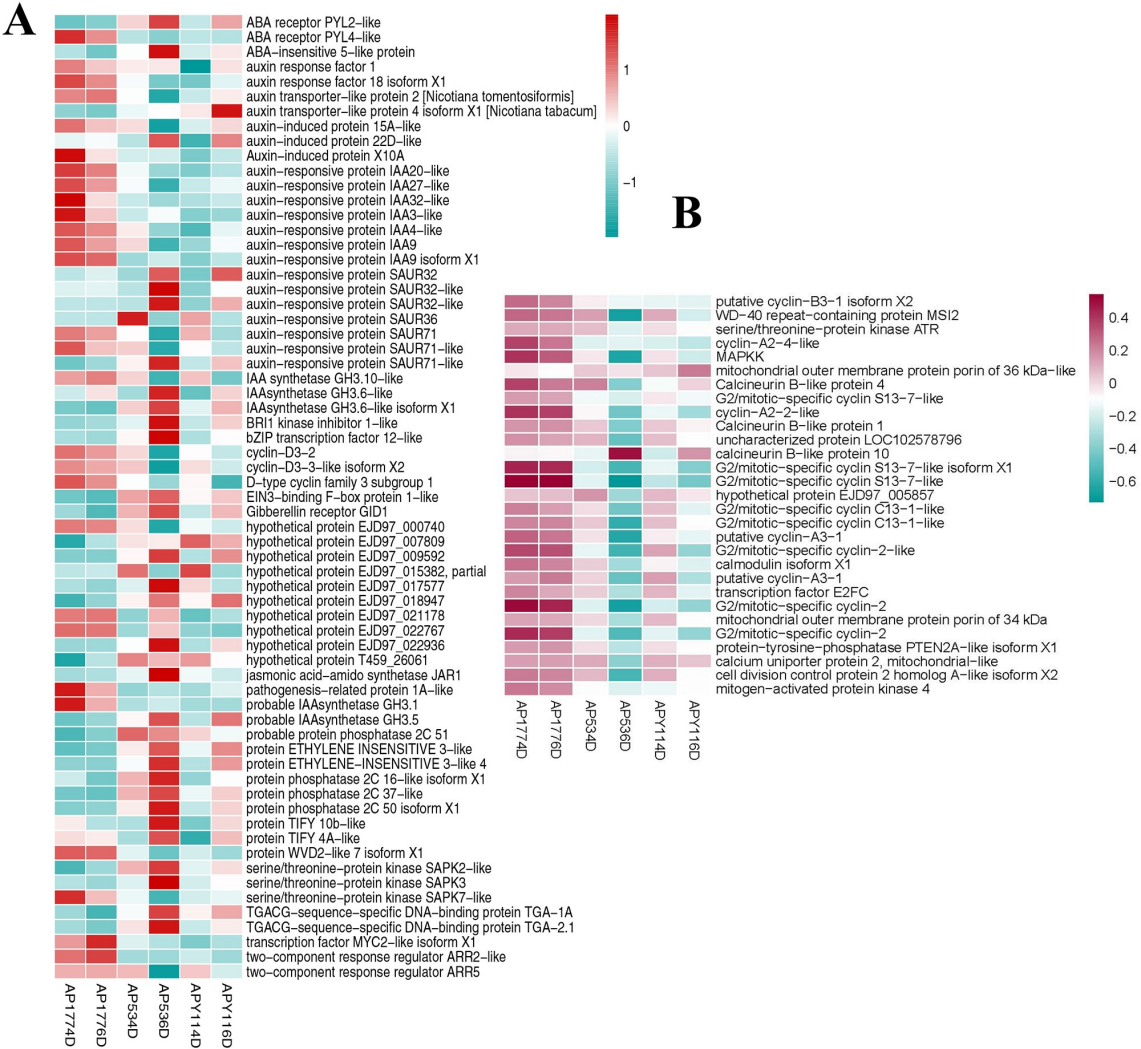
Expression patterns of differentially expressed genes involved in different pathways. Shown are genes related to: A. the plant hormone conduction pathway; and B. cellular senescence.

**Fig 5:**
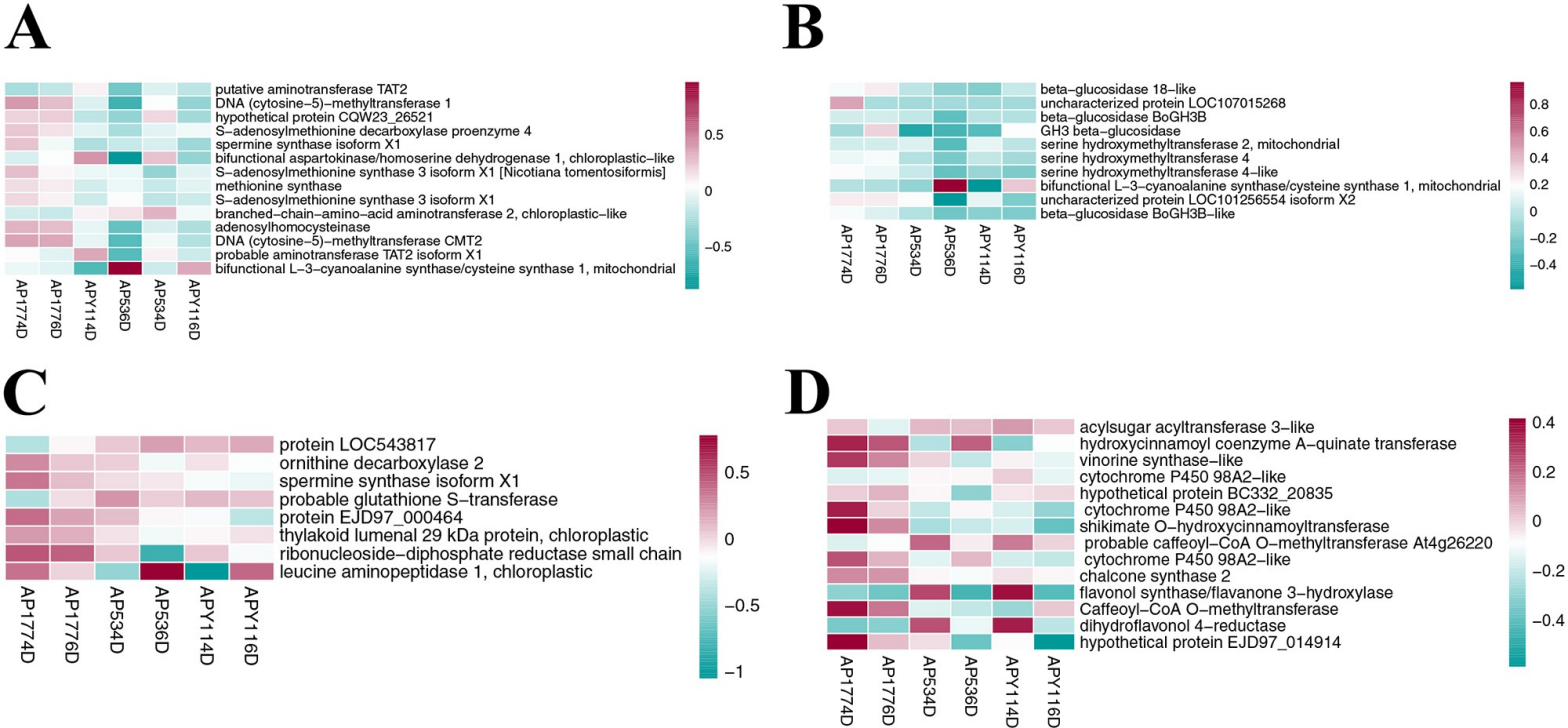
Expression patterns of differentially expressed genes involved in different pathways. Shown are genes related to: A. cysteine and methionine metabolism; B. cyanoamino acid metabolism; C. glutathione metabolism; and D. flavonoid biosynthesis.

**Fig 6:**
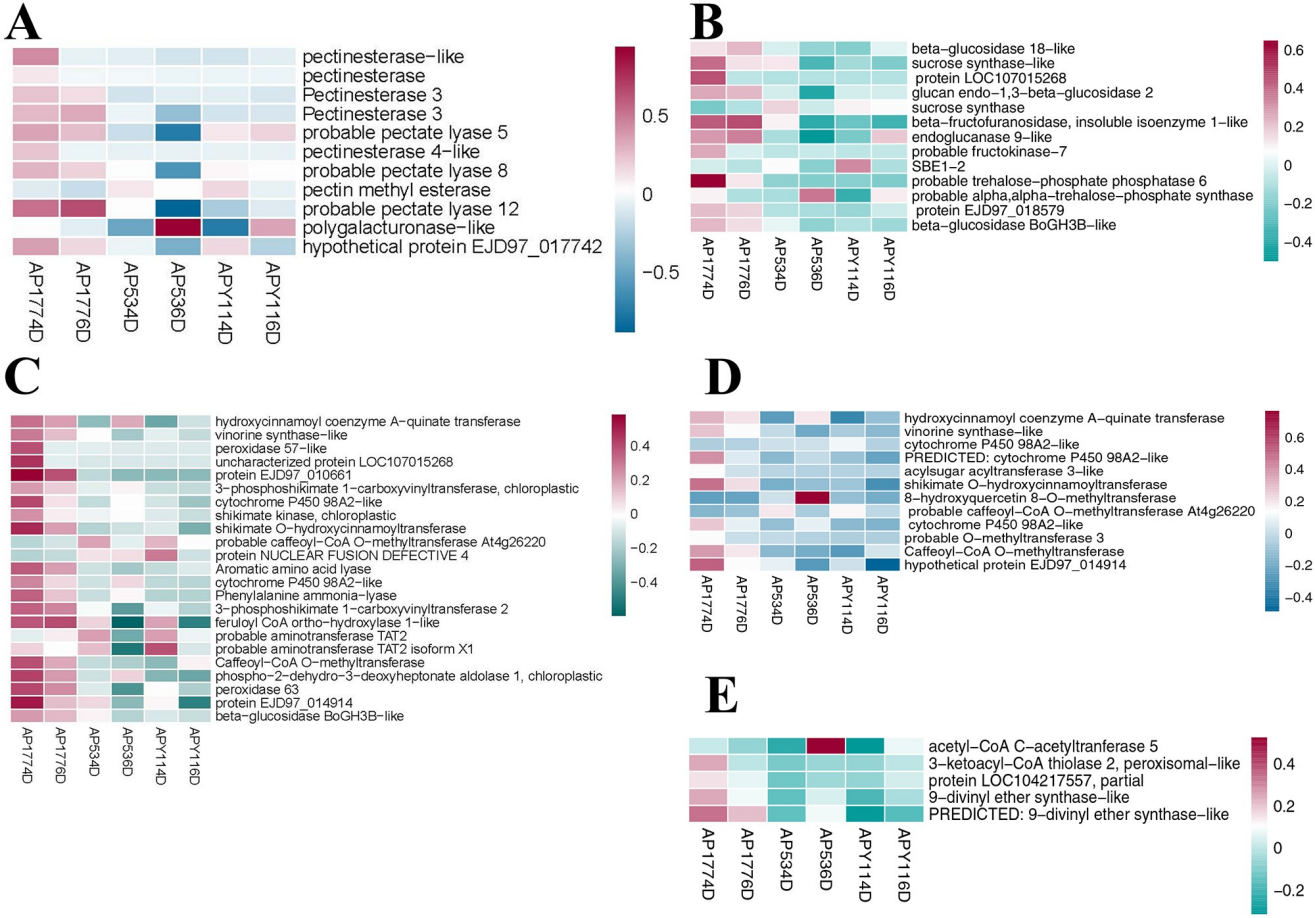
Expression patterns of differentially expressed genes involved in different pathways. Shown are genes related to: A. pentose and glucuronate interconversions; B. starch and sucrose metabolism pathways; C. phenylpropanoid biosynthesis; D. stilbenoid, diarylheptanoid, and gingerol biosynthesis; E. alpha-linolenic acid metabolism.

KEGG enrichment analysis was performed on the DEGs of AP177 vs AP53 and AP177 vs APY1 4 and 6 days after pollination (S4 Fig). The results showed that the DEGs were significantly enriched in plant hormone signal transduction, cellular senescence, flavonoid biosynthesis, and starch and cyanoamino acid metabolism in the four groups (Figs 4, 5A and 6A). Four days after pollination, the DEGs of AP177 vs AP53 and AP177 vs APY11 participated in 122 and 130 KEGG metabolic pathways, respectively, and 19 and 22 pathways were significantly enriched (uncorrected P-value < 0.05), respectively. Among them, pentose and glucuronate interconversions; flavonoid biosynthesis; stilbenoid; diarylheptanoid and gingerol biosynthesis; phenylpropanoid biosynthesis; glutathione metabolism; starch and sucrose metabolism; cellular senescence; relaxin signaling pathway; cyanoamino acid metabolism; alpha-linolenic acid metabolism; plant hormone signal transduction; cysteine and methionine metabolism; indole alkaloid biosynthesis; and chenylalanine, tyrosine, and tryptophan biosynthesis pathways were significantly enriched in both groups (S5, S4A and S4B Figs; Figs 5B, 5C and 6). Six days after pollination, the DEGs of AP177 vs AP53 and AP177 vs APY11 covered 140 and 123 KEGG metabolic pathways, respectively, with 25 and 8 significantly enriched pathways, among which plant hormone signal transduction, cellular senescence, flavonoid biosynthesis, and starch and cyanoamino acid metabolism pathways were significantly enriched in both groups (S4C and S4D Fig). Thus, these pathways are likely to be involved in the double fertilization process and contribute to the differences between self-fertilized and hybrid offspring.

### DEGs involved in plant hormone transduction and cellular senescence

Double fertilization in plants is a complicated biological process that is expected to integrate multiple signals. We identified DEGs involved in many pathways, partly confirming its complexity. Signal transduction pathways are known to play crucial roles in responding to low temperature stress. Through KEGG enrichment analysis, we found that hormone signaling pathways (plant hormone signal transduction) was significantly enriched in all pollination combinations, with a total of 66 genes that responded differently 4 and 6 days after pollination (Fig 4A). Among them, there were 26 auxin-related, 5 jasmonic acid-related, 5 ethylene-related, 1 gibberellin-related, 13 cytokinin-related, 3 salicylic acid-related, 1 brassinosteroid (BR)-related, and 12 abscisic acid-related genes (Fig 4A). In the auxin signal transduction pathway, 26 genes responded differently 6 days after pollination, among which, four genes (*EGP18001*, *EGP13566*, *EGP24068*, and *EGP12583*) were downregulated in the self-crossed and upregulated in the hybridized specimens. Sixteen genes (*EGP05905*, *EGP05353*, *EGP06582*, *EGP06526*, *EGP12698*, *EGP02442*, *EGP09809*, *EGP23892*, *EGP15918*, *EGP16416*, *EGP28299*, *EGP14225*, *EGP28710*, *EGP24015*, *EGP00757*, and *EGP29698*) were upregulated in the self-crossed and downregulated in the hybridized specimens. *EGP18974* was downregulated in the self-crossed and upregulated in the hybridized specimens 4 days after pollination, but was upregulated in the self-crossed and downregulated in the hybridized specimens 6 days after pollination. *EGP16886*, *EGP10981*, *EGP10983*, *EGP14643*, and *EGP00813* were upregulated in the self-crossed and downregulated in the hybridized specimens 4 days after pollination, but were downregulated in the self-crossed and upregulated in the hybridized specimens 6 days after pollination.

In the jasmonic acid signal transduction pathway, two genes responded differently 4 and 6 days after pollination, and three genes simultaneously responded differently 4 and 6 days after pollination (Fig 4A). *EGP26497* was downregulated in the self-crossed and upregulated in the hybridized specimens 4 and 6 days after pollination. *EGP06195* and *EGP05385* were upregulated in the self-crossed and downregulated in the hybridized specimens 4 and 6 days after pollination. *EGP01296* and *EGP06847* were upregulated in the self-crossed and downregulated in the hybridized specimens 4 days after pollination, but were downregulated in the self-crossed and upregulated in the hybridized specimens 6 days after pollination. These five genes may play an important role in double fertilization.

In the abscisic acid signal transduction pathway, twelve genes simultaneously responded differently 4 and 6 days after pollination (Fig 4A). *EGP12001* and *EGP24656* were upregulated in the self-crossed and downregulated in the hybridized specimens 4 and 6 days after pollination. *EGP26119*, *EGP22312*, *EGP06320*, *EGP06048*, *EGP11066*, *EGP09680*, *EGP24310*, *EGP01528*, *EGP09363*, and *EGP19130* were downregulated in the self-crossed and upregulated in the hybridized specimens 4 and 6 days after pollination. These twelve genes may play an important role in double fertilization.

Cellular senescence is involved in various stages of growth and death and was enriched in all pollination combinations in our study (Fig 4B). In this pathway, twenty-nine genes responded differently 4 and 6 days after pollination, among which twenty-seven genes simultaneously responded differently 4 and 6 days after pollination and two genes responded differently 4 and 6 days after pollination. The proportion of upregulated genes in the self-crossed specimens was higher than that of the hybridized specimens 4 and 6 days after pollination. Only *EGP10474* was downregulated in the self-crossed and upregulated in the hybridized specimens 4 and 6 days after pollination. The others were upregulated in the self-crossed and downregulated in the hybridized specimens 4 and 6 days after pollination. These twenty-seven genes may play an important role in double fertilization. *EGP19897* was upregulated in the self-crossed and downregulated in the hybridized specimens 4 days after pollination, but was downregulated in the self-crossed and upregulated in the hybridized specimens 6 days after pollination. *EGP24384* was downregulated in the self-crossed and downregulated in the hybridized specimens 4 days after pollination, but was upregulated in the self-crossed and downregulated in the hybridized specimens 6 days after pollination.

Methionine is a precursor of ethylene and cysteine plays a role in ethylene synthesis via methionine. Thus, the cysteine and methionine metabolism pathway is vital to plant growth and development. Through KEGG enrichment analysis, we found that cysteine and methionine metabolism was significantly enriched in all pollination combinations (Fig 5A). In this pathway, fourteen genes responded differently 4 and 6 days after pollination in the self-crossed and hybridized specimens, among which ten genes responded differently in the self-crossed and hybridized specimens 4 and 6 days after pollination and four genes responded differently 4 and 6 days after pollination. Only *EGP25958* was downregulated in the self-crossed and upregulated in the hybridized specimens 4 and 6 days after pollination. *EGP02776*, *EGP04256*, *EGP04413*, *EGP11067*, *EGP18416*, *EGP21477*, *EGP21624*, *EGP26284*, and *EGP26457* were upregulated in the self-crossed and downregulated in the hybridized specimens 4 and 6 days after pollination. *EGP27000* was upregulated in the self-crossed and downregulated in the hybridized specimens 4 days after pollination, but was downregulated in the self-crossed and upregulated in the hybridized specimens 6 days after pollination. *EGP00684*, *EGP14090*, and *EGP26835* were downregulated in the self-crossed and upregulated in the hybridized specimens 4 days after pollination, but were upregulated in the self-crossed and downregulated in the hybridized specimens 6 days after pollination.

Cyanoamino acid metabolism and glutathione metabolism pathways involve carbohydrate metabolism, which is related to carbohydrate metabolism in this study. Cyanoamino acid metabolism was significantly enriched in all pollination combinations (Fig 5B) and the glutathione metabolism pathway was enriched in the hybridized specimens 4 days after pollination (Fig 5C). Ten genes responded differently after pollination in the self-crossed and hybridized specimens in the cyanoamino acid metabolism pathway. *EGP00319*, *EGP03116*, *EGP05448*, *EGP13464*, *EGP24386*, *EGP26283*, *EGP32203*, and *EGP32205* were upregulated in the self-crossed and downregulated in the hybridized specimens 4 and 6 days after pollination. *EGP15078* was downregulated in the self-crossed and upregulated in the hybridized specimens 4 days after pollination, but was upregulated in the self-crossed and downregulated in the hybridized specimens 6 days after pollination. *EGP27000* was upregulated in the self-crossed and downregulated in the hybridized specimens 4 days after pollination, but was downregulated in the self-crossed and upregulated in the hybridized specimens 6 days after pollination. Eight genes responded differently after pollination in the self-crossed and hybridized specimens in the glutathione metabolism pathway. *EGP10782*, *EGP11067*, *EGP18110*, *EGP28187*, and *EGP31824* were upregulated in the self-crossed and downregulated in the hybridized specimens 4 and 6 days after pollination. *EGP00485* and *EGP18102* were downregulated in the self-crossed and upregulated in the hybridized specimens 4 and 6 days after pollination. *EGP33526* was upregulated in the self-crossed and downregulated in the hybridized specimens 4 days after pollination, but was downregulated in the self-crossed and upregulated in the hybridized specimens 6 days after pollination.

### Expression of genes involved in metabolism and biosynthesis

Through KEGG enrichment analysis, we found that flavonoid biosynthesis was significantly enriched in all pollination combinations (Fig 6A). The proportion of upregulated genes was higher in the self-crossed specimens than in the hybridized specimens 4 and 6 days after pollination. Fifteen genes responded differently 4 and 6 days after pollination in the self-crossed and hybridized specimens. *EGP01180*, *EGP03132*, *EGP12728*, and *EGP31016* were downregulated in the self-crossed and upregulated in the hybridized specimens 4 and 6 days after pollination. *EGP27514* was downregulated in the self-crossed and upregulated in the hybridized specimens 4 days after pollination, but was upregulated in the self-crossed and downregulated in the hybridized specimens 6 days after pollination. The other genes were upregulated in the self-crossed but downregulated in the hybridized specimens 4 and 6 days after pollination.

### Transcription factors involved in the regulation of double fertilization

**Fig 7:**
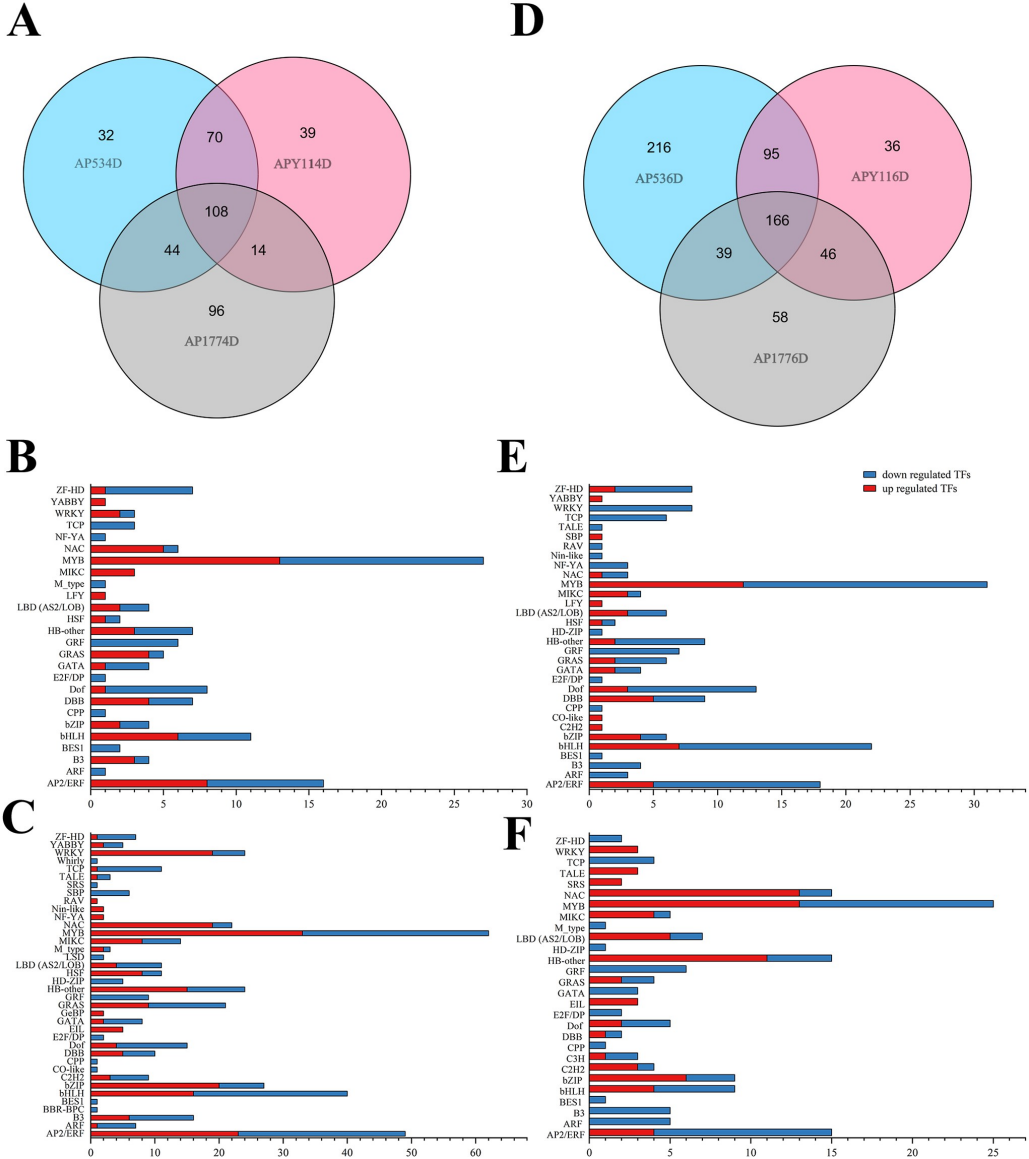
Different expression patterns of transcription factors. A. Venn diagram of transcription factors 4 days after pollination; B-C. family name and gene number of differentially expressed transcription factors 4 days after pollination; D. Venn diagram of transcription factors 6 days after pollination; E-F. family name and gene number of differentially expressed transcription factors 6 days after pollination.

Transcription factors play important roles in plant development, especially in double fertilization. The Plant-TFDB was used to predict and analyze the transcription factors of all annotated DEGS by Blast, and 1683 transcription factors were identified. The highest proportion of transcription factors was in the ERF family (10.34%), followed by MYB (8.20%), MYB-related (7.61%), bHLH (7.31%), and B3 super (6.36%) families (S5 Fig). Among them, 108 transcription factors were detected 4 days after pollination (Fig 7A). Four days after pollination, the MYB super family encompassed the most upregulated transcription factors in AP53 and APY11 (Fig 7B and 7C). Six days after pollination, 166 transcription factors were detected in all three pollination combinations (Fig 7D). Six days after pollination, the MYB super family encompassed the most downregulated transcription factors in AP53 and APY11 (Fig 7E and 7F). The MYB super family represented the most downregulated transcription factors in AP53 (Fig 7E), and the MYB and NAC super families represented the most downregulated transcription factors in APY11 (Fig 7F).

### Validation of RNA-Seq-based DEGs by qRT-PCR

**Fig 8:**
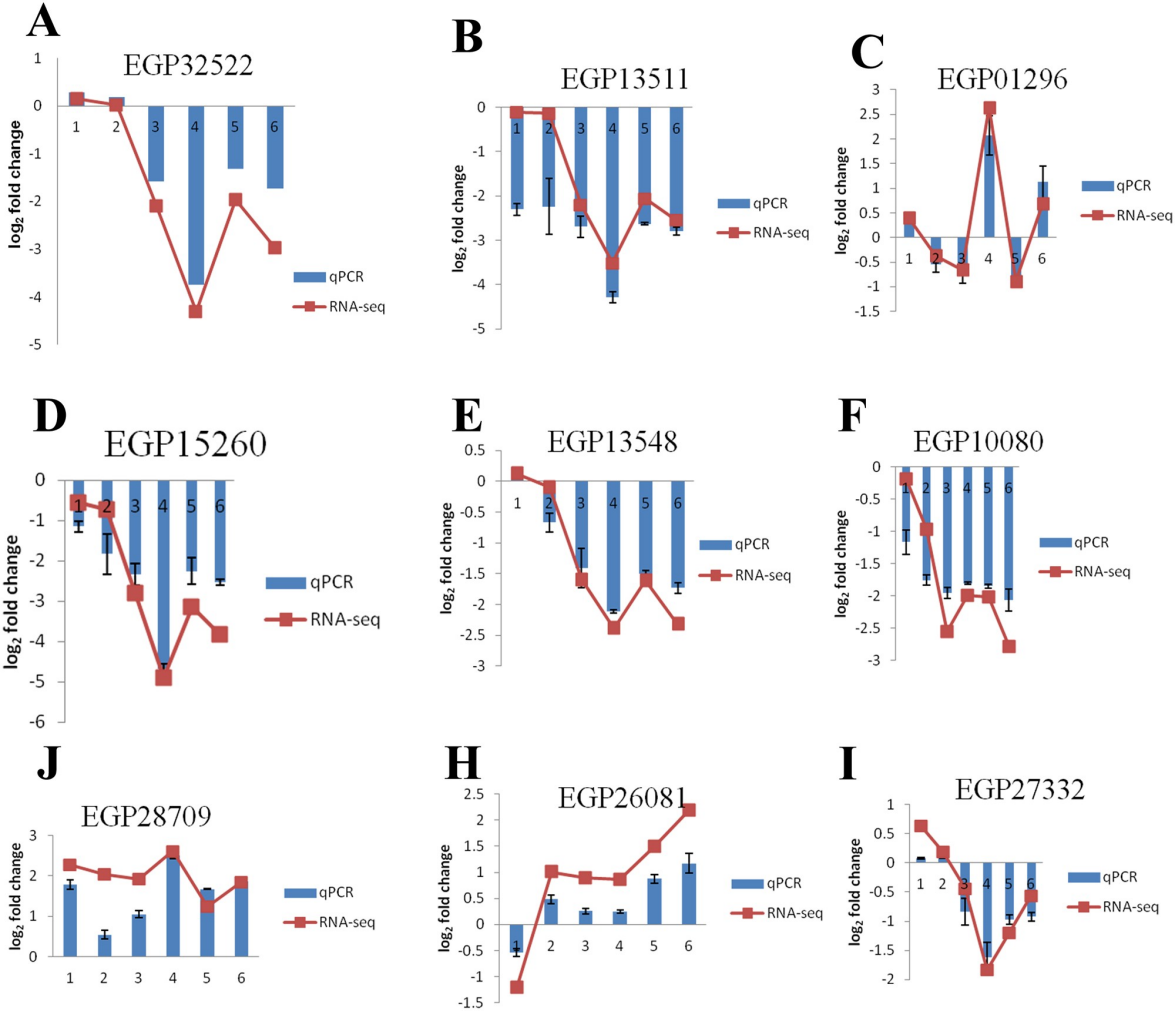
qRT-PCR analysis of differentially expressed genes. On each abscissa, 1–6 represents AP1774D, AP1776D, AP534D, AP536D, APY114D, and APY116D, respectively.

We selected nine DEGS and used qRT-PCR to analyze the expression patterns and verify the results of RNA-Seq (Fig 8). The expression trend of these nine genes in qRT-PCR was basically consistent with that of RNA-Seq, indicating high accuracy of the RNA-Seq results (Fig 7). EGP15260, EGP13511, and EGP10080 were downregulated 4 and 6 days after pollination. EGP28709 was upregulated 4 and 6 days after pollination, which was consistent with the RNA-Seq results.

## Discussion

Distant hybridization is an important method for breeding improvement and germplasm innovation. However, cross-incompatibility increases the difficulty of this breeding technique. Cross-incompatibility may result in abnormal pollen germination and pollen tube behavior, low fruit-setting rates, and a decreased number of single fruit seeds [29, 30]. We found that *S*. *melongena* could be reciprocally crossed with *S*. *aethiopicum*, and through histological observation, we found no evident difference, but different parents produced significant differences in seed numbers. These seed number differences are consistent with those reported in previous studies [12, 31], which may be related to differences in cross-incompatibility [32]. However, we found no report on the histological causes of these differences. Potential reasons for the differences require further study, possibly extending the observation time post-pollination.

Previous studies have shown that plant hormones are correlated with plant pollination and fertilization, embryo development, and seed formation [33, 34]; ethylene can suppress seed development and auxin-related hormones can promote it [35]. Auxins are mainly produced in seeds [36–38]. Considering our data on the expression of genes in the auxin signal transduction pathway, we only found five genes downregulated in the self-crossed and upregulated in the hybridized specimens, while sixteen genes were upregulated in the self-crossed and downregulated in the hybridized specimens. This indicates that high expression of auxin-related genes may be beneficial to distant hybridization and results in significantly higher seed numbers per fruit. A previous study on embryogenesis in *Arabidopsis* showed that the auxin signaling pathway, which was associated with gene activity, is more prevalent in early developmental stages when the post-fertilization sporophytic program is initiated [39]. Abscisic acid has been associated with stress-induced senescence and is known to have a stimulatory effect on abscission [38]. In our data on the expression of genes related to the abscisic acid signal transduction pathway, only two were upregulated in the self-crossed and downregulated in the hybridized specimens, while ten genes were downregulated in the self-crossed and upregulated in the hybridized specimens. This indicates that a lower expression of abscisic acid-related genes may be detrimental to distant hybridization, resulting in significantly lower seed numbers per fruit. Our finding was consistent with former research results [35, 40–42]. Intensive studies of auxin-related and abscisic acid-related DEGs may be beneficial to understanding the regulatory mechanisms underlying double fertilization and providing a theoretical basis for developing high seed-yield hybrids.

Distant hybridization was also enriched in flavonoid biosynthesis; stilbenoid, diarylheptanoid and gingerol biosynthesis; phenylpropanoid biosynthesis; glutathione metabolism, starch and sucrose metabolism; cyanoamino acid metabolism; alpha-linolenic acid metabolism; cysteine and methionine metabolism; indole alkaloid biosynthesis; and chenylalanine and tyrosine and tryptophan biosynthesis when compare with the self-crossed combination (P<0.05); most of these genes were downregulated in the hybridization when compared with the self-crossed combination. These pathways mainly involved amino acid metabolism, organic acid metabolism and sugar metabolism, and phenylalanine metabolic pathways. The results showed that distant hybridization could mainly change the metabolism of amino acids, organic acids, and substances related to glucose metabolism. On the one hand, changes in the glycolytic pathway, TCA cycle, and phenylpropionic acid metabolism would improve antioxidant capacity and resistance to the stress caused by foreign pollen. On the other hand, distant hybridization would not be able to produce sufficient resources to meet fertilization and seed development demand, resulting in fewer seed numbers than with the self-crossed combination [43]. Thus, there is ample evidence to indicate that seed and fruit set are regulated by these metabolites [44, 45].

As regards the AP2/ERF family of transcription factors, some of them are involved in protection against cell death [46]. Their upregulation in apple lateral fruit seeds further supports the contention that the ethylene response is activated [38]. Our data on the expression of the AP2/ERF family genes support this, where most genes were downregulated in the hybridization compared with the gene levels in the self-crossed combination. The AP2/ERF family of transcription factors may be related to double fertilization.

In summary, RNA-Seq analysis created a comprehensive view of the participation of several pathway genes in the self-crossed and hybridized combinations. The self-crossed and hybridization of *Solanum* were affected by various factors such as sucrose metabolism, and signal transduction, hormones, transcriptional regulation, and other factors can affect the degree of seed abortion. The main causes of abortion include resource constraints, sibling competition, inbreeding decline, genetic load, and hormone regulation. It may also consist of a complex regulatory network in which multiple factors work together. The results of our study contribute to a better understanding of the molecular mechanisms involved in the double fertilization of interspecific hybridization. However, more research on "omics", specifically the overall analysis of the correlation between related genes and metabolic pathways, is needed in order to understand the differences further. Nevertheless, our study represents an important step for elucidating the biological pathways involved in interspecific hybridization and more studies are being conducted to verify the function of these possible genes and their involvement in this process.

## Supporting information

S1 Fig Comparative analysis of gene timing expression patterns in self-crossed and hybridization ovaries. A. Analysis of differential gene timing expression patterns after 4 and 6 days in unpollinated and post-pollinated 177; B. Analysis of differential gene timing expression patterns after 4 and 6 days in unpollinated and post-pollinated 53; C. Analysis of timing expression patterns of differential genes at 4 and 6 days in unpollinated and post-pollinated Y11. The number above the image indicates the pattern, and the number in brackets indicates the number of genes in the pattern; each image represents an expression pattern.
(DOCX)

S2 Fig Subcluster 2 GO analysis. A. 177; B. 53; C. Y11.
(DOCX)

S3 Fig DEG GO enrichment analysis. A. DEG GO enrichment between AP1774D and AP534D; B. DEG GO enrichment between AP1774D and APY114D; C. DEG GO enrichment between AP1776D and AP536D; D. DEG GO enrichment between AP1776D and APY116D.
(DOCX)

S4 Fig DEG KEGG enrichment analysis. A. DEG KEGG enrichment between AP1774D and AP534D; B. DEG KEGG enrichment between AP1774D and APY114D; C. DEG KEGG enrichment between AP1776D and AP536D; D. DEG KEGG enrichment between AP1776D and APY116D.
(DOCX)

S5 Fig Analysis of predicted transcription factors. (DOCX)

S1 Table Statistical table of charge data. (XLSX)

S2 Table Assembly result evaluation. (XLSX)

S3 Table Primer information. (XLSX)
