# Supplementary material for: Comparative transcriptome analysis provides insights into the molecular mechanism underlying double fertilization between self-crossed Solanum melongena and that hybridized with Solanum aethiopicum
Source: PLoS One. 2020 Aug 6;15(8):e0235962. doi: 10.1371/journal.pone.0235962 (PMC7410197; doi:10.1371/journal.pone.0235962)
Supplement: S1 Fig — A. Analysis of differential gene timing expression patterns after 4 and 6 days in unpollinated and post-pollinated 177; B. Analysis of differential gene timing expression patterns after 4 and 6 days in unpollinated and post-pollinated 53; C. Analysis of timing expression patterns of differential genes at 4 and 6 days in unpollinated and post-pollinated Y11. The number above the image indicates the pattern, and the number in brackets indicates the number of genes in the pattern; each image represents an expression pattern. (DOCX) [file pone.0235962.s001.docx]

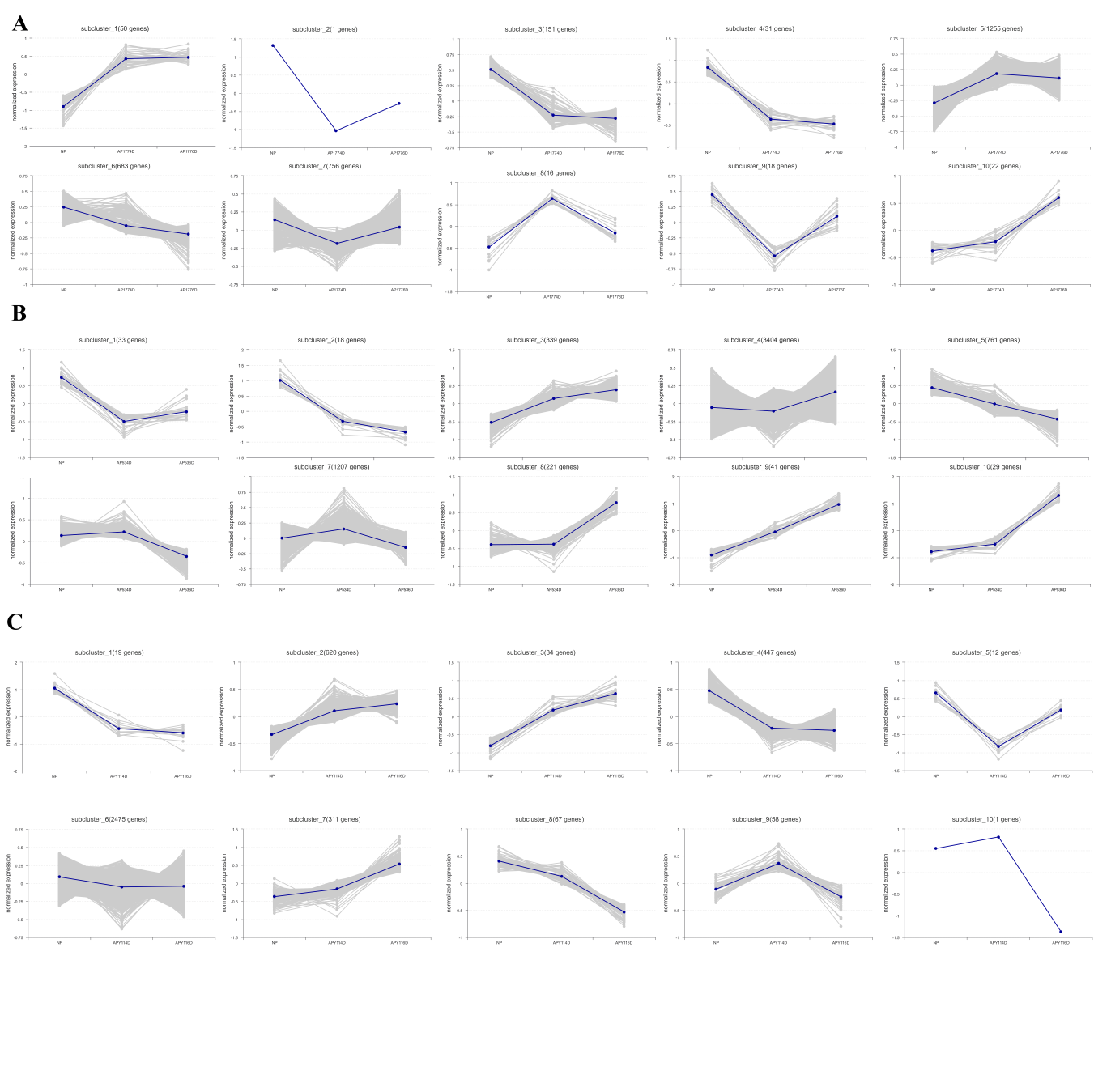


The number at the top of each image indicates the pattern, and the number in brackets indicates the number of genes in the pattern.Each picture represents a mode of expression.

Fig. S1 A :The timing sequence expression pattern analysis of differential genes before pollination and after pollination 4 and 6 days of 177 ;B The timing sequence expression pattern analysis of differential genes before pollination and after pollination 4 and 6 days of 53 ;C The timing sequence expression pattern analysis of differential genes before pollination and after pollination 4 and 6 days of Y11
