# Supplementary figures and images for: Comparative transcriptome analysis provides insights into the molecular mechanism underlying double fertilization between self-crossed Solanum melongena and that hybridized with Solanum aethiopicum
Source: PLoS One. 2020 Aug 6;15(8):e0235962. doi: 10.1371/journal.pone.0235962 (PMC7410197; doi:10.1371/journal.pone.0235962)

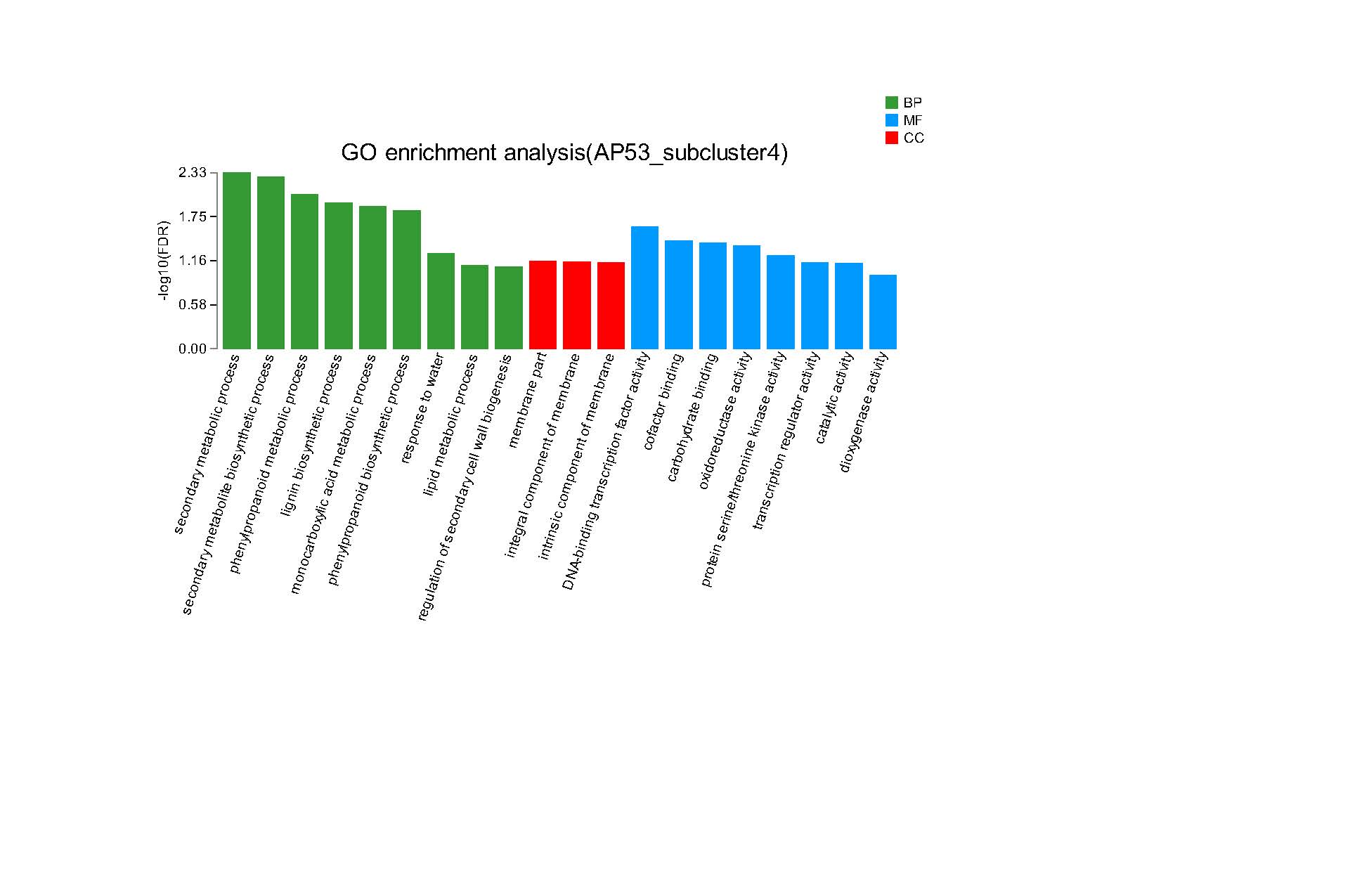


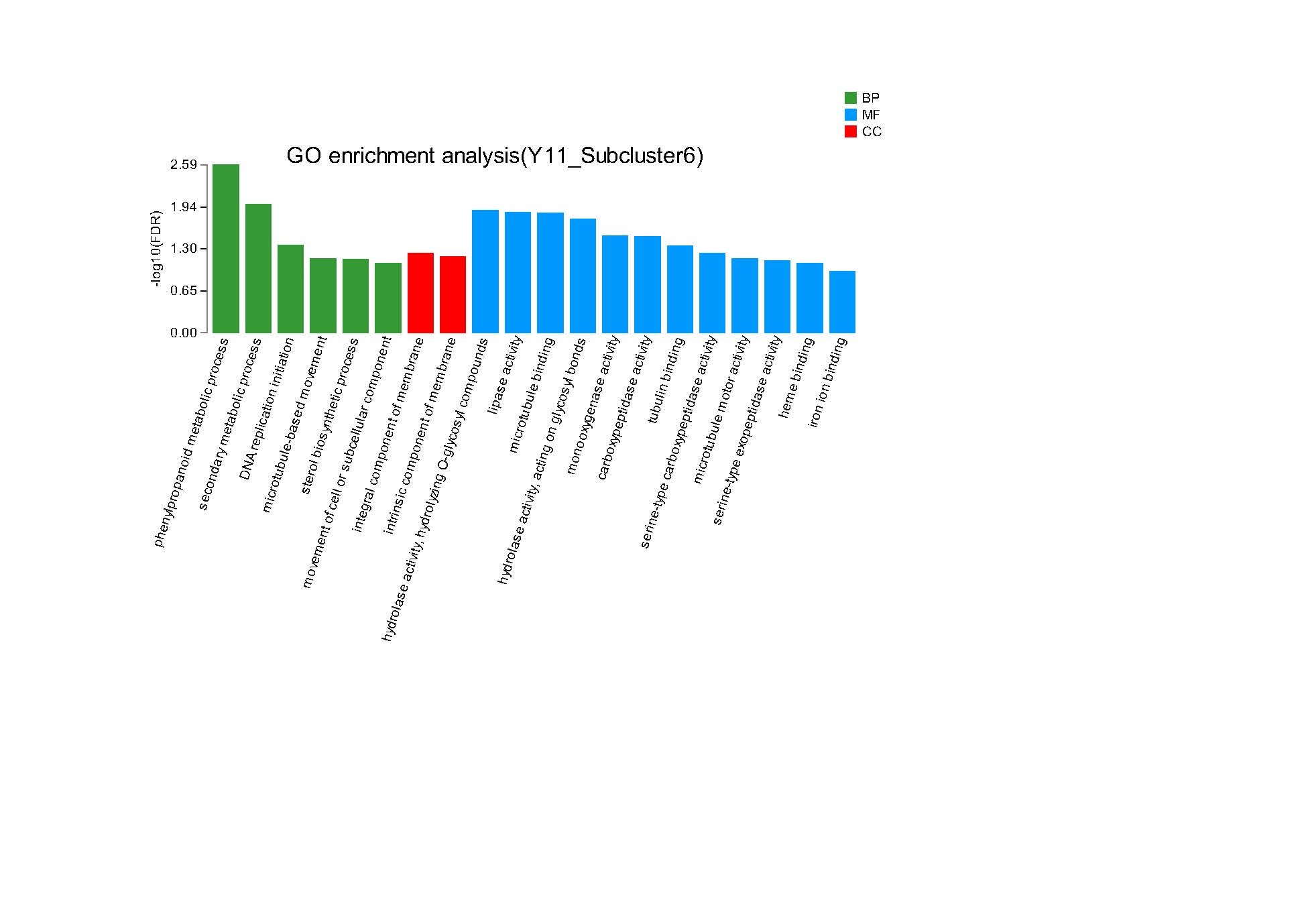

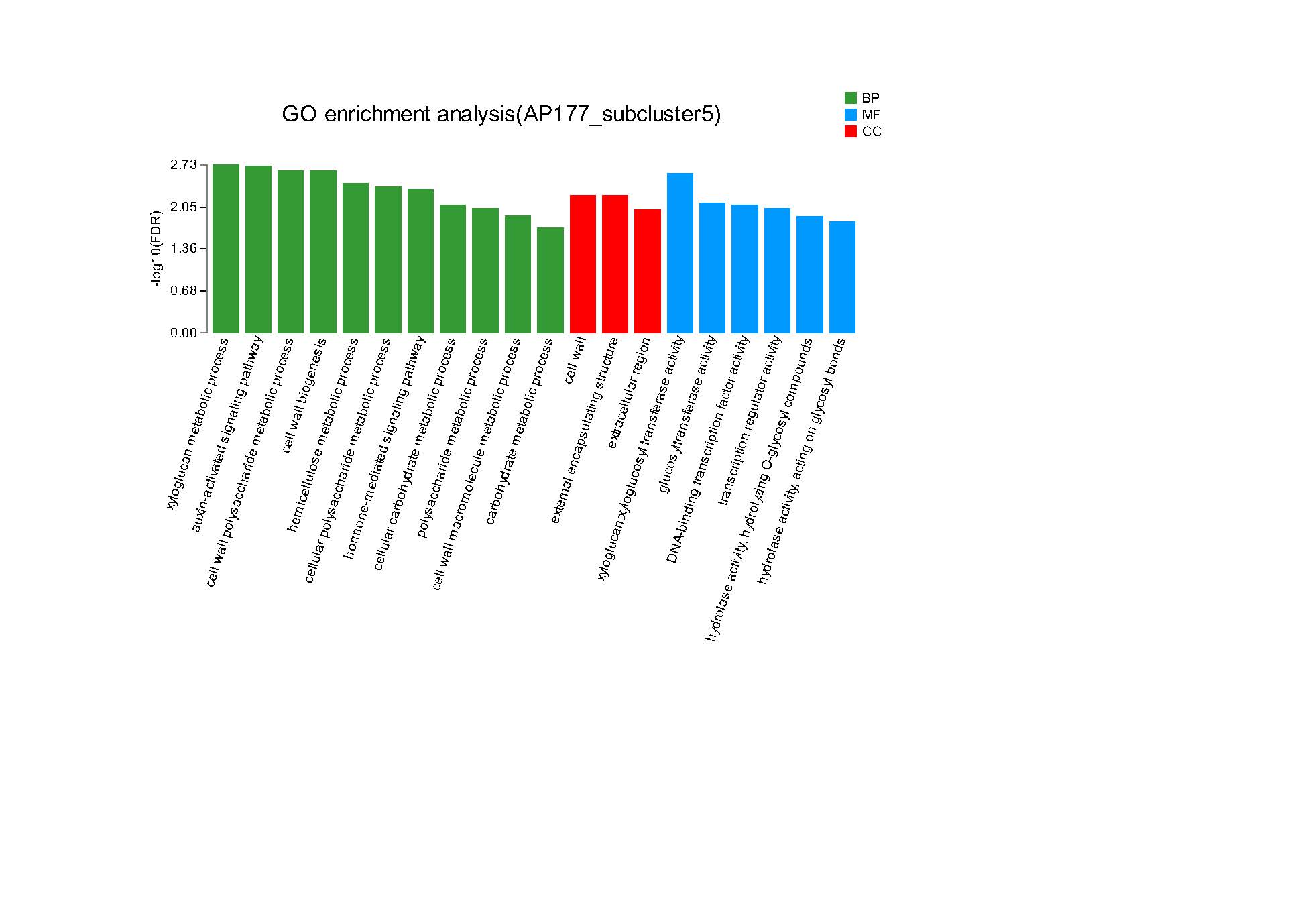


Fig S2 subcluster GO analysis: 177 ; 53 ; Y11

Supplement: S2 Fig — A. 177; B. 53; C. Y11. (DOCX) [file pone.0235962.s002.docx]

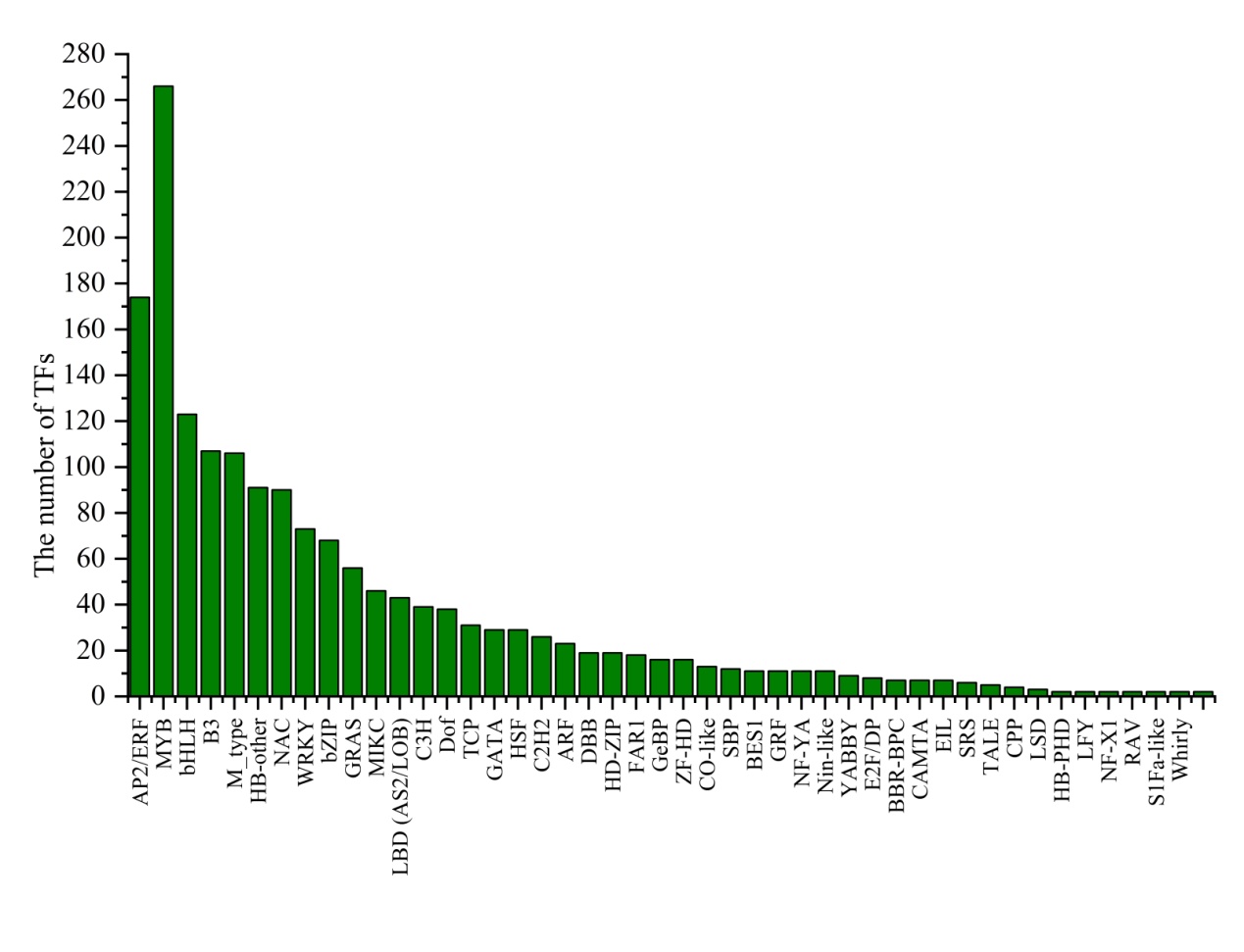


Fig S5: The analysis of predicted transcription factors

Supplement: S5 Fig — (DOCX) [file pone.0235962.s005.docx]
