# Supplementary material for: Comparative transcriptome analysis provides insights into the molecular mechanism underlying double fertilization between self-crossed Solanum melongena and that hybridized with Solanum aethiopicum
Source: PLoS One. 2020 Aug 6;15(8):e0235962. doi: 10.1371/journal.pone.0235962 (PMC7410197; doi:10.1371/journal.pone.0235962)
Supplement: S3 Fig — A. DEG GO enrichment between AP1774D and AP534D; B. DEG GO enrichment between AP1774D and APY114D; C. DEG GO enrichment between AP1776D and AP536D; D. DEG GO enrichment between AP1776D and APY116D. (DOCX) [file pone.0235962.s003.docx]

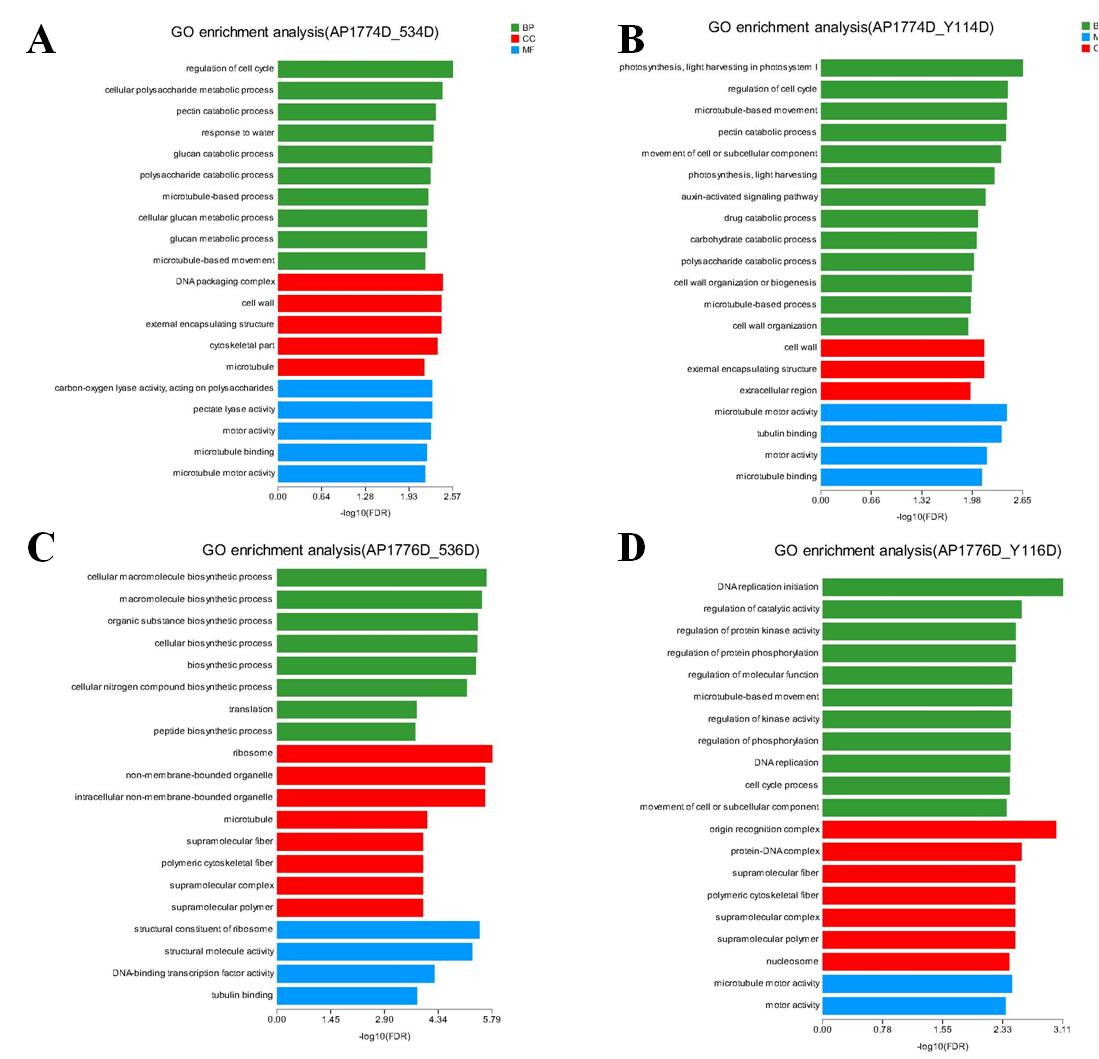


Fig S3 A: DEGs GO enrichment between AP177 4D and AP53 4D; B: DEGs GO enrichment between AP177 4D and APY11 4D; C: DEGs GO enrichment between AP177 6D and AP53 6D;D: DEGs GO enrichment between AP177 6D and APY11 6D
